# Occupational safety and health criteria for responsible development of nanotechnology

**DOI:** 10.1007/s11051-013-2153-9

**Published:** 2013-12-07

**Authors:** P. A. Schulte, C. L. Geraci, V. Murashov, E. D. Kuempel, R. D. Zumwalde, V. Castranova, M. D. Hoover, L. Hodson, K. F. Martinez

**Affiliations:** 1 National Institute for Occupational Safety and Health, Centers for Disease Control and Prevention, 4676 Columbia Parkway, MS C-14, Cincinnati, OH 45226 USA; 2 Hassett Willis and Co., Washington, DC USA

**Keywords:** Risk assessment, Ethics, Risk management, Regulation, Toxicology, Environmental and health effects

## Abstract

Organizations around the world have called for the responsible development of nanotechnology. The goals of this approach are to emphasize the importance of considering and controlling the potential adverse impacts of nanotechnology in order to develop its capabilities and benefits. A primary area of concern is the potential adverse impact on workers, since they are the first people in society who are exposed to the potential hazards of nanotechnology. Occupational safety and health criteria for defining what constitutes responsible development of nanotechnology are needed. This article presents five criterion actions that should be practiced by decision–makers at the business and societal levels—if nanotechnology is to be developed responsibly. These include (1) anticipate, identify, and track potentially hazardous nanomaterials in the workplace; (2) assess workers’ exposures to nanomaterials; (3) assess and communicate hazards and risks to workers; (4) manage occupational safety and health risks; and (5) foster the safe development of nanotechnology and realization of its societal and commercial benefits. All these criteria are necessary for responsible development to occur. Since it is early in the commercialization of nanotechnology, there are still many unknowns and concerns about nanomaterials. Therefore, it is prudent to treat them as potentially hazardous until sufficient toxicology, and exposure data are gathered for nanomaterial-specific hazard and risk assessments. In this emergent period, it is necessary to be clear about the extent of uncertainty and the need for prudent actions.

## Introduction

The responsible development of nanotechnology is a goal of many organizations worldwide (e.g. Royal Society and Royal Academy of Engineering 2004; NIOSH 2005; Jacobstein 2006; CEST 2008; Tomellini and Giordani 2008; Luigi 2009; Nanocyl 2009; NNI 2011; Forloni 2012; VCI 2012; BASF 2013; BIAC 2013). Ideally, the concept of responsible development of nanotechnology implies that there are criteria against which to evaluate development. The focus of those criteria is the prevention of harm to people, and the environment. Workers are the first people exposed to the potential hazards of any new technology including nanotechnology, since they are involved in the research, development, manufacture, production, use, recycling, and disposal of nanomaterials or products containing nanomaterials. Workers often have the highest exposure, which may occur early in the development of a technology when hazards and risks are uncertain. If exposure to nanomaterials harms workers, then nanotechnology is not being responsibly developed. For these reasons, occupational safety and health is the cornerstone of responsible nanotechnology development (Maynard and Kuempel 2005; Schulte and Salamanca-Buentello 2007; Seaton et al. 2010). Anticipating and preventing harm to consumers from products containing nanomaterials is also part of responsible development, as is anticipating how nanomaterials might adversely impact the environment.

There is a moral imperative for worker protection which is of paramount importance, i.e., workers have rights to a safe work environment (Gewirth 1986). Thus, safety of work was recognized as a basic human right by the 2008 Seoul Declaration on Safety and Health at Work (ILO 2008). These rights bring commensurate responsibilities for employers and government authorities to protect workers from harm as fully as is reasonably possible. These responsibilities have been codified in laws and regulations such as the Occupational Safety and Health (OSH) Act, the Mine Safety and Health (MSH) Act, the Toxic Substances Control Act (TSCA) in the United States, and similar legislation and guidance worldwide (e.g., WHO 1994; BAuA 2007; Nanosafe 2008; ISO 2009; Japan NIOSH 2009; Pelley and Saner 2009; Bayer 2010; Murashov et al. 2011; Nakanishi 2011a, b).

Underlying the criteria for responsible development of nanotechnology is the need to be proactive in taking steps to limit exposure of workers, consumers, and the environment to nanomaterials before actual risks are fully understood (Kreider and Halperin 2011). These criteria are not new, but build on basic OSH principles that should be applied to nanotechnology at the early stages in its development. While evidence-based risk assessment and management are the ideals, often action must be taken with less than strong evidence. Moreover, the ultimate component of responsible development is to reduce hazards and risks to the extent feasible and to communicate with, and engage, affected parties (workers) in the management of risks. In this article, the occupational safety and health criteria for responsible development of nanotechnology are defined and their implications are described. These criteria can be considered at the business enterprise and societal levels. Ideally, the criteria would be developed at the societal level (by government agencies, trade, and professional associations, unions, non-governmental organizations (NGOs), insurers, scientists) first and then promoted for use at the business enterprise level (employers, suppliers, business customers). In reality, nanotechnology products were in commerce before criteria for responsible development were in place. That does not mean that there were no applicable societal expectations. The whole history of societal response to hazardous materials provided a framework to initially address the products of nanotechnology. Already in place, as mandated by the OSH Act of 1970 (Public Law 91–596) in the United States, as well as guidance developed by other countries and organizations (e.g., EU Directive 89/336/EEC and Directive 98/21/EC), was the concept that the employer must provide a safe and healthy workplace. Definitions of “safe” and “healthy” for nanomaterials build on experience gained in the 20th century on addressing worker hazards and risks from exposure to fine dusts and powders in various industries such as pigment, pharmaceutical, nuclear, and pesticide manufacturing (Higgins 1917; Dressen et al. 1938; Cook 1945; Sargent and Kirk 1988; Maiello and Hoover 2011). Also, in place were validated risk management practices for controlling fine dusts and powders coming out of the fields of aerosol science, industrial hygiene, exposure assessment, toxicology, and engineering (Hinds 1999).

A body of knowledge developed over the past 100 years shows that small particles can, on an equal mass basis, be more hazardous than larger ones (Driscoll 1996; IOM 2000; Zhang et al. 2000, 2003; Brown et al. 2001; Duffin et al. 2002; Oberdörster et al. 2007; Seaton et al. 2010). Thus, it was known, before, engineered nanomaterials entered commerce that incidental nanoparticles (e.g., welding and diesel fumes) could be carcinogenic when inhaled (Oberdörster and Yu 1990; Heinrich et al. 1995; Antonini 2003); that small aerosol pollutants were linked to respiratory and cardiovascular risks (Dockery et al. 1993; Pope et al. 2002); and that certain “legacy produced” nanomaterials such as ultrafine titanium dioxide, carbon black, and fumed silica were respiratory hazards (Reuzel et al. 1991; Oberdörster et al. 1994; Gardiner et al. 2001; Merget et al. 2002). Clearly too, there also was extensive literature that larger (microscale) inhaled particles are known respiratory hazards (e.g., silica, coal dust) (NIOSH 2002, 2011a). The relationship between particle lung dose and adverse lung effects (e.g., pulmonary inflammation or lung tumors in rats) has been observed to be nonlinear for poorly-soluble low toxicity particles, with no clear particle size threshold (NIOSH 2011b). Nonetheless, for many decision-makers at the business or societal levels, how to define the responsible approach for the safe development of engineered nanomaterials was unclear; and, in some cases, it remains so today, in the second decade of commercialization. This is despite the fact that precautionary guidance has been promulgated by authorities since the mid-2000s (EU-OSHA 2002; Roco and Bainbridge 2003; Hett 2004; HSE 2004; NNI 2004; NIOSH 2005; SCENIHR 2005; ISO 2007; Safe Work Australia 2010a; OSHA 2013).

## Criteria for responsible development

Five criterion actions (Table 1) may be considered that demonstrate responsible development from an occupational safety and health perspective. These include: (1) anticipate, identify, and track potentially hazardous nanomaterials in the workplace; (2) assess workers’ exposures to nanomaterials; (3) assess and communicate hazards and risks to workers; 4) manage occupational safety and health risks; and (5) foster the safe development of nanotechnology and the realization of its societal and commercial benefits. These criteria can be assessed at the business enterprise and societal levels. They are based on various influences, including the history of occupational safety and health in the 20th century; the anticipatory work of various researchers and officials (Ashford 1976; Samuels 1986; Colvin 2002; Roco 2003; Aitken et al. 2004; Maynard and Kuempel 2005; Maynard 2006; Murashov and Howard 2008; Tomellini and Giordani 2008; Howard 2011; Roco et al. 2011; Murashov and Howard 2013); the ethical framework described by Schulte and Salamanca-Buentello (2007); and the practices of governmental agencies [e.g., the National Institute for Occupational Safety and Health (NIOSH)], corporations, and labor organizations worldwide conducting research or developing risk management guidance. The criteria also build on the 1983 and 2009 risk assessment paradigms by the U.S. National Research Council (NRC 1983) of the National Academies of Science; the Rio Conference of 1992 (UN 1992); the reports by the National Academy of Engineering (2004) and the Royal Society and Royal Academy of Engineering (2004); the principles of tiered toxicological screening (Oberdörster et al. 2005); and the Nano Risk Framework (2007).

**Table 1:** Occupational safety and health criteria that demonstrate responsible development of nanotechnology

Criteria	Business enterprise responsibility	Societal responsibility
Anticipate, identify, and track potentially hazardous nanomaterials in the workplace	Identify nanomaterials in the workplace Conduct toxicologic research Take precautionary (prudent) approaches	Issue anticipatory guidance Conduct toxicologic research Issue hazard guidance and control
Assess workers' exposures to nanomaterials	Measure exposure	Provide guidance on metrics, sampling methods, and analysis
Assess and communicate hazards and risks to workers	Conduct hazard and risk assessments Communicate hazard and risk information to workers Train workers in safe handling techniques	Conduct hazard and risk assessments, including quantitative estimates Communicate risk information to employers, unions, workers, other agencies, and the public
Manage occupational safety and health risks	Manage workplace risks from nanomaterials Control exposures Monitor workers exposure and health	Include many partners to develop governance strategies Issue guidance on workplace risk management OELs (occupational exposure limits) Engineering controls and PPE (personal protective equipment) Medical surveillance
Foster the safe development of nanotechnology and the realization of societal and commercial benefits	Protect workers from any harm from nanomaterials Convey the degree of uncertainty known about risks Acknowledge hazards Support precautionary approaches Document the effectiveness of controls	Convey the degree of certainty about hazards and risks Conduct research to address uncertainties Demonstrate the effectiveness of controls Address relationship between occupational and environmental hazards Work globally Support education and scientific literacy

**Fig. 1:**
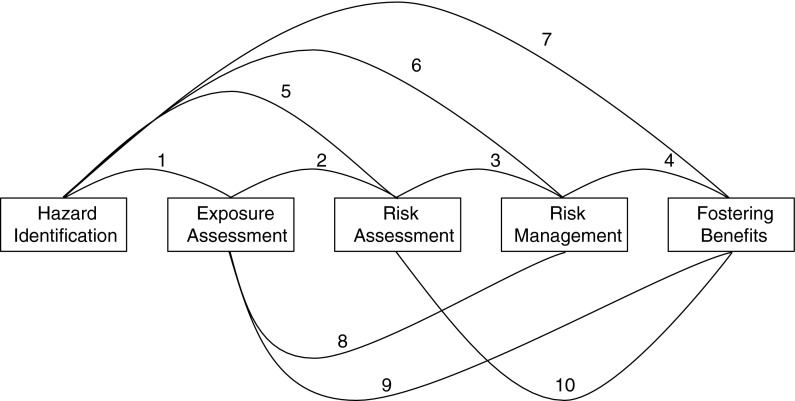
Interrelation of criteria for responsible development of nanotechnology

Integration of knowledge from all these related criteria is important, because (Fig. 1; Table 2) actions under one criterion may influence actions under the others (European Commission 2005; Kuempel et al. 2012a; Savolainen 2012). Hazard information generally will drive much of the downstream actions, but each criterion can influence all the others. Responsible development requires integrated action among decision-makers addressing each criterion. At the business level, this may include activities of employers along the supply and value chains, and lifecycle of products. At the societal level, this can include activity of regulators, trade and professional associations, insurers, and NGOs, in endeavors supporting each criterion and all of them taken together.

**Table 2:** Relationship between occupational safety and health criteria for responsible development of nanotechnology

Relationship^a^		Implications
1.^b^	HI ↔ EA	Provides priorities for exposure assessment
2.	EA ↔ RA	Component factor in risk assessment provides priorities for exposure assessment
3.	RA ↔ RM	Informs risk management
4.	RM ↔ FB	Minimizes worker risks and enhances societal acceptance
5.	HI ↔ RA	Component factor in risk assessment; provides priorities for toxicology study
6.	HI ↔ RM	Triggers risk management
7.	HI ↔ FB	Identifying worker hazards useful for identifying consumer and environmental hazards
8.	EA ↔ RM	Assessing exposures is critical in controlling them
9.	EA ↔ FB	Identifying risk of exposures to workers provides information needed for effective risk management
10.	RA ↔ FB	True depiction of risks and risk management decisions to minimize risk enhances societal acceptance

^a^ *HI* hazard identification (anticipate, identify and track potentially hazardous nanomaterials in the workplace), *EA* exposure assessment (assess workers exposures to nanomaterial), *RA* risk assessment (assess and communicate hazards and risks to workers), *RM* risk management (manage occupational safety and health risks), *FB* foster benefits (foster the safe development of nanotechnology and the realization of societal and commercial benefits)

^b^Number pertains to linkages in Fig. 1

The overarching driver in responsible development of nanotechnology and for each criterion is establishing responsibility for workplace safety and worker exposure. At the business level, the responsibility for a safe and healthy workplace is that of the employer. Early in the commercialization of nanotechnology, many employers indicated that they did not know much about the hazards, risk, exposure, and control of nanomaterials. This uncertainty about the hazards and risks could have resulted in many employers not undertaking the necessary responsibility to protect their workers (Ponce Del Castillo 2013). This uncertainty about risks prompted government agencies to provide guidance on these issues. Workers and their representatives also have responsibilities to advocate for safe and healthy workplaces, to encourage and participate in risk management efforts at the business level, and to advocate for protective guidance at the societal level (CalOSHA 2013; HSE 2013). Society as a whole has the responsibility to support and empower employers, workers, unions, governments, and others in meeting their responsibilities. In addition, the public must be knowledgeable and engaged in deliberations considering new technologies, especially in regards to potential health risks that might be associated with this technology.

### Anticipate, identify, and track potentially hazardous nanomaterials in the workplace

Due diligence and legal mandates require employers to be aware of hazards to which their employees could be exposed and all hazards present in facilities that they control (including nanomaterials as well as other chemical or physical hazards). When there is uncertainty about the nature, degree, and extent of hazards of nanomaterials, it is incumbent on employers to know what nanomaterials are in their workplaces, to identify processes where exposures can occur, and to support studies to determine the bioactivity of the nanomaterials. This is not always a simple matter for employers who might unknowingly be using intermediaries or product ingredients containing nanomaterials. Recent data have suggested that important information with regard to nanomaterials is not being included on current Safety Data Sheets (Safe Work Australia 2010b; Eastlake et al. 2012; Lee et al. 2012). In addition, with the advent and rollout of the globally harmonized system (GHS) of classification and labeling of chemicals, it is unclear how nanomaterials will be identified, classified, and labeled. Nevertheless, employers must consider the potential hazards of materials they manufacture or procure. When a concern exists, the employer should utilize existing exposure control guidance or seek expertise on implementing appropriate control measures. Critical to assessing the potential health risk to the material is the need to keep updated on new and changing hazard information.

At the societal level, anticipation and identification of hazards requires government agencies and other organizations to identify what nanomaterials are being widely made and used, their hazard potential, and how to control them (OECD 2010; Safe Work Australia 2010a). This information needs to be communicated clearly and should describe the levels of certainty of the existing data and where there are gaps in the data. Government authorities and manufacturers are responsible for applying resources to test various nanomaterials, in order to better proscribe and issue hazard assessment and control guidance. Toxicological research is essential for responsible development of nanotechnology (Oberdörster et al. 2005; Oberdörster et al. 2007; Stone et al. 2013). Toxicological research first showed that nanomaterials such as ultrafine titanium dioxide (TiO_2_) and carbon nanotubes (CNTs) can cause adverse respiratory effects in animals, indicating the potential to cause respiratory disease in workers (Heinrich et al. 1995; Shvedova et al. 2005; Dankovic et al. 2007; Oberdörster et al. 2007). This hazard identification was not an easy task. For example, with CNTs, the challenge was not merely to expose animals to atmospheres containing CNTs, but also to consistently generate aerosols of dimensions and characteristics in animal studies that had relevance to potential worker exposures (McKinney et al. 2009).

Toxicological research is the basis for hazard identification. The responsible development of nanotechnology requires continued investment in such research. For due diligence under TSCA in the US or registration, evaluation, authorization, and restriction of chemical substances (REACH) in the European community, employers will need to continue to invest in toxicological research on nanomaterials. Linking toxicology testing to hazard determination is not new to the global chemical industry. Moving to the nanoscale has revealed new or heightened biological activity driven by size and physico-chemical properties, and employers will need to continue to explore the role of these parameters on toxicity. Better understanding of the correlation between physico-chemical properties and toxicity will facilitate assessment of hazards for new nanomaterials and the design of safer nanomaterials.

At the societal level, responsible development requires investment in toxicological assessment of widely used nanomaterials and in development of predictive models allowing estimation of hazards of new nanomaterials, as well as preventing particularly hazardous nanomaterials from entering into commerce (Oberdörster et al. 2005; Xia et al. 2010; Clark et al. 2011; Bonner et al. 2013; Winkler et al. 2012). Such efforts also need to be accompanied by communication of hazards found in such assessments. Additionally, responsible development involves anticipating future nanomaterials development and applications for commercialization. This includes consideration of more complex and active nanomaterials (Subramanian et al. 2010; Murashov et al. 2012).

Standardized characterization criteria and validated assays and algorithms are needed to classify engineered nanomaterials by the nature and degree of hazard. Given the broad diversity of nanomaterial types, this will require basic research on what properties of nanomaterials can be linked to toxic effects. Tools to make categorical estimates of toxicity, such as various alternative testing strategies, quantitative structure–activity relationship (QSAR) models, computational toxicology, and bioinformatics, need to be applied to untested materials with similar properties and used as the basis for initial risk management decisions (Kuempel et al. 2012b; Nel et al. 2013; Stone et al. 2013).

Knowledge of hazards has been increased by international collaborations such as those supported by the Organisation for Economic Co-operation and Development (OECD), United States–European union bilateral efforts, World Health Organization (WHO), International Organization for Standardization (e.g., ISO TC 229), and the various agreements between nations. Participation in these collaborations is an important aspect of responsible development.

If correct decisions are to be made about hazards, risks, and control of nanomaterials, the scientific research needs to be conducted in these areas. Whereas, results from first-generation short-term toxicity testing were used to anticipate hazards from a small number of nanomaterials and implement exposure control measures, there is ultimately a need for standardized approaches for toxicological evaluation, setting priorities for toxicity testing, and long-term (chronic health effects) investigations (Oberdörster et al. 2005; Savolainen 2012; Bonner et al. 2013; Stone et al. 2013).

It is not informative enough to just identify hazards; there also is a need to know who is being exposed to them, at what exposure concentrations, and how exposure is affected by changes in job tasks. When the degree of hazard has not been ascertained, the general guidance of government agencies is to treat candidate nanomaterials in their workplaces as if they are potential hazards until a higher level of certainty about the presence or degree of hazard is available (Philbrick 2010; Schulte et al. 2012).

### Assess workers’ exposure to nanomaterials

Critical in assessing and managing risks is the measurement of exposures to nanomaterials (Ramachandran et al. 2011). This is a complex endeavor, especially this early in the natural history of engineered nanomaterials, when what constitutes appropriate exposure metrics is not clear (Brouwer et al. 2012; Ostraat et al. 2013). Nonetheless, the earliest guidance has indicated that particle mass/volume of air can be a useful metric for measuring airborne exposures to nanomaterials. Since the first issuance of the “NIOSH Approaches to Safe Nanotechnology” at the NanOEH2 symposium in Minneapolis in 2005, government guidance on how to assess worker exposure and implement risk management strategies continues to be refined and updated as new information is obtained (NIOSH 2009a).

Assessing exposures in the workplace is the employer’s responsibility, however, it is incumbent on government agencies and other organizations to assess in a general sense, the extent to which worker exposures are controlled and that guidance is provided on measurement approaches [e.g., the European Union framework 7 nanodevice project (http://www.nano-device.eu/index.php?id=123)]. The basis for this guidance, like that for identifying hazard potential, will be an evolving body of knowledge that should be continually evaluated. Continued efforts to identify hazards and determine workplace exposures are necessary to develop and update risk management guidance. Exposure is a critical factor that drives risk, and hence assessment and management of the risks. In order to minimize exposures, employers need to know and should assess what exposures actually occur, as well as their magnitude and background conditions. To adequately define occupational exposure to nanomaterials, information obtained by workplace environmental monitoring could be complemented by biological monitoring strategies that assess exposure by all routes.

Exposure assessment is important for other efforts that lead to responsible development of nanotechnology. These include helping to identify populations at risk, both in terms of actual exposures or exposure potential, and linking exposure to adverse effects in epidemiological studies (Schulte et al. 2009; Dahm et al. 2012; Riediker et al. 2012). Exposure assessment data can also serve as a sampling frame for the formation of exposure registries (i.e., lists of workers with actual or potential exposure) that can be used in future epidemiologic and health surveillance studies. Exposure assessment data are also used in conducting risk assessments and in setting occupational exposure limits (OELs) (Schulte et al. 2010). Sharing exposure data by making it publicly available would facilitate this task and should be considered a part of responsible development of nanotechnology.

### Assess and communicate hazards and risks to workers

Risk assessments by definition are evaluations to predict risks when adequate data are available, although complete data are often lacking. Risk assessments are based on various assumptions and may include a high degree of uncertainty. This is especially true for nanomaterials at this early stage in their development. Yet, when adequate data are available for risk analysis, it is prudent to use such data as the basis of initial risk management decisions, while identifying and acknowledging the uncertainties.

Ultimately, the type of risk management practices needed to protect workers will depend on the extent of the risks (Schulte and Ringen 1984; Jonsen 1991; NRC 2009; Gibson et al. 2012). Risk is a probabilistic concept that depends on both the hazard and the exposure. Characterizing the reliability and the uncertainty in risk estimates will be important in risk communication and management. Employers can perform qualitative risk assessments by identifying where and to what extent exposures to nanomaterials occur, or could occur among workers in their facilities. In addition, quantitative risk assessments (QRAs) allow for estimation of risks based on empirical data. For QRAs such as those conducted by authoritative organizations, the process includes the extrapolation of toxicology data from laboratory animal studies given the limited availability of epidemiological data. For airborne nanoparticles, this involves normalization of the lung burdens associated with adverse effects in animals to estimate the equivalent human lung burdens from worker exposure information (Kuempel et al. 2006; 2012b).

Evaluating what data and information are needed to support decision-making is important in risk assessment. Although conducting QRAs on individual nanomaterials is useful, it is likely that adequate toxicological information on which to base the assessment will be available for only a small number of nanomaterials. Since there are currently many more nanomaterials than there is hazard or exposure information, it may be that risk assessment and the resulting exposure limits will focus on categories of nanomaterials (OECD 2007; Kuempel et al. 2012b). A categorical approach allowing for a relatively rapid assessment of a large number of engineered nanomaterials will be especially useful in developing risk management policies in the early decades of nanotechnology commercialization.

While hazard communication to workers is codified in laws in some countries (OSHA 2012), risk communication is less often included (HSA 2005), but is an ethical responsibility based on the right-to-know (Yale Law Journal 1981; Schulte and Ringen 1984; Jonsen 1991). The right of workers to know risk information is widely accepted and the duty of employers to communicate risk information derives from it. In some cases, explicit risk information is not available to employers, but there is information on components of risk—hazard and exposure. If employers communicate nanomaterial hazard and exposure information, they are conducting a basic form of risk communication. Hazard and risk communication related to nanomaterials are critical aspects of the responsible development of nanotechnology (CEST 2008; Schulte and Salamanca-Buentello 2007). Hazard and risk communication should be conducted by employers to nanomaterial workers, and also by manufacturers to their downstream users who may then use such information in their communications with workers.

It is not enough to merely assess risks; it is important that employers, government agencies, and other stakeholder organizations communicate what is known about the risks (Shatkin et al. 2010). Ideally, risk communication should be two-directional rather than one-directional (Ponce del Castillo 2013). The appropriate risk communication will depend on the risk perception of the intended audiences, particularly workers, and the extent to which they can participate in discussions of the risks and training about them (Kulinowski and Lippy 2012). Risk communication and risk management are most effective when workers and employers are empowered to act on those risks.

### Manage occupational safety and health risks

While employers are responsible for the management of risks, they often require guidance from authorities on appropriate risk management practices. This is especially true for nanomaterials, for which the knowledge base is limited and for small employers with limited expertise or resources. The general guidance from authorities has been to be aware of where nanomaterials are used and, as a precautionary measure, to control exposures as much as reasonably achievable. Early guidance from authorities was that free, unbound nanomaterials follow the laws of classic aerosol physics and that exposures can be controlled by the same approaches historically used for fine dusts, powders, and gases. As more knowledge was accrued, guidance included benchmark, provisional, or recommended single-substance exposure limits (BSI 2007; Nakanishi 2011b; NIOSH 2011b, 2013a, b; van Broekhuizen et al. 2012). Other precautionary guidance came from applications of proposed and existing regulations to nanomaterials, such as, for manufacturers to submit risk management plans for carbon nanotubes under significant new use rules (SNUR) to the US Environmental Protection Agency (EPA) under TSCA (EPA 2013). In addition, efforts to develop voluntary consensus standards for safe handling of nanomaterials in the workplace (e.g., ISO TC 229), were an early illustration of responsible development (ISO 2009). It is the responsibility of employers to use the best available guidance as the basis for controlling exposures in the workplace (including training workers), and workers have the responsibility to cooperate with employers in carrying out risk management processes.

Various commentators have discussed the need for regulation of nanomaterials, similar to regulation of other workplace hazards (Pelley and Saner 2009; Ling et al. 2012; van Broekhuizen et al. 2012). Regulations can be critical to the implementation of good risk management practices and a keystone for responsible development of nanotechnology (Murashov et al. 2011). Although some existing general regulations may be considered to address nanomaterials, a good example of a specific approach is the recent international standard adopted in Canada for occupational exposure to engineered nanomaterials (Canadian Standards Association 2012). Voluntary international standards can be a major force in the responsible development of nanotechnology and are more likely to be in place than specific national standards as evidenced by the ISO Standards on nanotechnology (Murashov and Howard 2008; Murashov and Howard 2013). Authoritative OSH recommendations from NIOSH and other agencies or organizations provide research and health-based criteria for promoting workplace health and safety (e.g., NIOSH 2009a, 2011b, 2013a).

The general basis for managing risks from hazards, including the potential hazards of nanomaterials, is to follow the hierarchy of controls (Peterson 1973; NIOSH 2013a, b). One means of implementing the hierarchy of controls, in light of uncertainties about the hazards of nanomaterials, is by hazard and control banding approaches (Naumann et al. 1996; NIOSH 2009b; Ostiguy et al. 2010; Brouwer 2012). Some efforts in this regard are already in effect, and their continued refinement is an important aspect of responsible development of nanotechnology (Paik et al. 2008; ANSES 2010). A hazard and control banding approach can be an alternative for controlling exposures when there is insufficient information for evidence-based OELs. Additionally, an important component of the application of control banding is verification of the performance and efficacy of controls for protecting workers’ health (Jones and Nicas 2006), as well as precaution in the application of these controls when health hazard data are limited (Schulte and Salamanca-Buentello 2007).

The most effective level of the hierarchy of controls is to eliminate or design out hazards (Schulte et al. 2008b). This can be accomplished for some nanomaterials by modifying specific physico-chemical parameters of the material. The idea is that by modifying the functionality of the nanomaterials, the commercial utility of the material can be maintained while potential toxicity is reduced or mitigated. Various organizations and government agencies have been exploring this approach (http://cnse.albany.edu/Outreach/NIOSHPresentations.aspx). Responsible development of nanotechnology requires continued investment in this area.

At the societal level, responsible development of nanotechnology involves the development of national and international partnerships, and OELs. The US and the EU have supported biannual bilateral conferences on occupational safety and health issues beginning in 2009; nanotechnology was a focal topic (US–EU 2012). In 2012, a set of overarching principles to guide research, guidance, legislation, and practice were developed under this US–EU partnership (Table 3). The criteria described in this article are consistent with those principles.

**Table 3:** Overarching principles to guide research, guidance, legislation, and practice involving nanotechnology

The health of workers should not be harmed by their work with nanomaterials
Globally harmonized definitions for engineered nanomaterials are needed
Transparency and traceability are essential to inform workers and employers if engineered nanomaterials are used in workplaces and where exposure may occur
Hazard and risk assessments must be performed to inform exposure control decisions for nanomaterials to which workers may be exposed
Emerging and enabling nanotechnology should apply “safe by design” principles to materials and processes to engineer out the hazardous or toxic potentials of new engineered nanomaterials as a best practice to protect workers and the environment
Early warning systems need to be developed to monitor workers’ health
Well-established industrial hygiene practices are appropriate to address nanotechnology hazards and risks
If occupational exposure limit values are not available for specific nanomaterials, a precautionary approach should be applied
Harmonized exposure assessment measurements and control strategies need to be developed for nanomaterial processes
Workers have the right to participate in developing risk management practices involving nanomaterials in the workplace

Adapted from the draft US–EU 7th joint conference on occupational safety and health, topic 1: nanotechnology at the workplace, Brussels, 11–13 July 2012. http://www.euusosh.org/

Various organizations and authorities have published OELs for nanomaterials in terms of categorical, provisional, or specific values (BSI 2007; NIOSH 2011b, 2013a, b; van Broekhuizen et al. 2012). In terms of regulatory frameworks, the promulgation of SNURs under TSCA by the EPA illustrates how employers can be required to address potential occupational safety and health concerns when using specific types of nanomaterials. Efforts to apply REACH provisions to nanomaterials are also under way. Ultimately, developing nanotechnology responsibly will require governments to work together and promote coordination and cooperation (Falkner and Jaspers 2012).

Risk management programs (e.g., evaluation of exposures, implementation of exposure controls, training, medical surveillance) for nanomaterials should be seen as part of an overall occupational safety and health program for any company or workplace producing or using nanomaterials (Schulte et al. 2008a), including those along the supply and value chains. In addition, for nanomaterials, as for many other substances in production and use, there is limited hazard information and often there are no specific OELs. Responsible development of nanomaterials also includes consideration of nanomaterials in the context of managing other workplace hazards.

An important aspect of risk management is the medical surveillance of nanotechnology workers (NIOSH 2009c). Medical surveillance allows for the identification of workers who exhibit signs and systems of adverse effects resulting from exposures to be adequately protected (Trout and Schulte 2010). For many nanomaterials, the health endpoint of interest for medical surveillance is not known, and hence only generalized medical surveillance is warranted (NIOSH 2009c). However, as new information is generated, more specific guidance for medical surveillance will be developed and should be implemented, as was recommended for workers exposed to carbon nanotubes and nanofibers (NIOSH 2013a, b).

In addition to medical surveillance, the potential long-term health experience of workers exposed to nanomaterials needs to be assessed through epidemiologic research and workplace exposure characterization studies. This is a difficult challenge since the nanomaterial workforce is widely distributed, exposed to a large number of different materials, and the appropriate health endpoints have not yet been consistently defined (Schulte et al. 2009; Riediker et al. 2012). In the short-term, cross-sectional studies using biomarkers may be the best approach; ultimately prospective and retrospective cohort studies will be needed (Li and Nel 2011; Liou et al. 2011; Schulte and Trout 2011; Riediker et al. 2012). Additionally as more hazard information becomes available, it may be useful to consider the value of registries of exposed workers in various sectors (Boutou-Kempf et al. 2011; Schulte et al. 2011). Such registries would allow more efficient identification of larger study populations, and may reduce the burden of epidemiology studies in the workplace.

Worker training is a key component in risk management and an indicator of responsible development of any technology (Kulinowski and Lippy 2012; Ponce del Castillo 2013). Although control of workplace exposures is the responsibility of the employer, training workers is integral to risk communication and management. Employers must train workers on workplace hazards and job tasks that may expose them to nanomaterials; on routes of exposure and methods used for controlling exposures; and on the use of respiratory protection and good work practices. Workers should also be informed about the potential health risks from exposure to nanomaterials and the possible need for medical surveillance (NIOSH 2009c). If nanotechnology is to be responsibly developed, worker protection has to be woven into codes of conduct, corporate responsibility pronouncements, and third-party certification schemes. Examples of such proactive efforts have illustrated how they can lead to worker protection (Nano Risk Framework 2007; Nanocyl 2009; Luigi 2009; BASF 2013; IG-DHS 2013; TÜV SÜD Industrie Service GmbH 2013; Verband der Chemischen Industrie 2012).

Continued dialog on risk management between nations and among stakeholders is needed. The international dialog on responsible research and development of nanotechnology is a firm foundation on which to build this dialog (Tomellini and Giordani 2008). Critical to the implementation of good risk management practices is the need to form partnerships among stakeholders to achieve common approaches to perceiving and controlling risks. Engaged stakeholders—such as corporations, trade associations, unions, nongovernmental organizations, insurance organizations, scientists, academic organizations, and government agencies—need to advocate for responsible development, and particularly for worker protection from potential adverse effects of nanomaterials.

### Foster the safe development of nanotechnology and the realization of its societal and commercial benefits

**Fig. 2:**
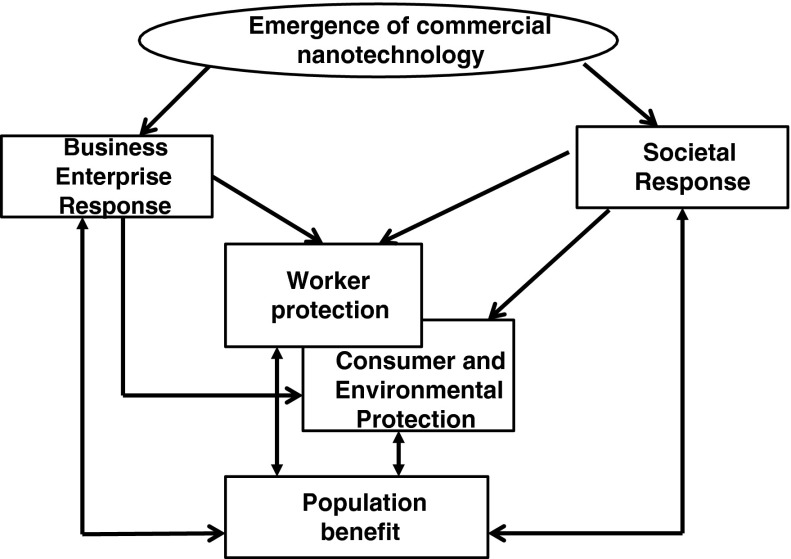
Pathways for responsible development of nanotechnology

At the societal level, responsible development of nanotechnology can provide benefits to workers and the rest of the population (Fig. 2) (Roco et al. 2011). Both the general population and the nanomaterial workforce may benefit from the diffusion of nanotechnology and nanoscience, but social and commercial benefits do not take precedence over worker safety and health. As part of the general population, workers can receive the projected societal benefits of nanotechnology (Roco 1997). These benefits may include good high paying jobs, and innovative products that address critical societal problems in materials, health, transportation, energy, and pollution. In addition to those societal gains, the workforce also benefits from nanotechnology research where nanotechnology-enabled products, such as nanotechnology-enabled sensors for detecting hazardous agents, nano-enhanced protection equipment, and nanomaterials, that are safer than traditional chemicals, have been developed to help ensure a safe work place. When workers are protected, the entire population benefits, because workers are part of it, and burdens on the population resulting from lack of protection are minimized. Others in the population are more receptive to new technologies when they see that precautions are taken, and workers are not harmed by nanomaterials (Hansen et al. 2008; Pidgeon et al. 2008; Savolainen 2012). Conversely, if the population sees that workers are harmed by nanomaterials, then it will be more resistant to products containing nanomaterials. Critical in this dynamic is the level of knowledge and certainty about protection and harm. Consequently, if employers, government agencies, or scientists are not clear in communicating the level of uncertainty about hazards and risks, or if they over-depict or under-depict those hazards and risks, then this may lead to an adverse reaction toward nanotechnology by the general population (Berube 2006; Tannert et al. 2007). If employers’ or authorities’ investments to address uncertainty are not adequate or timely, then this too can impede the development of the technology (Hansen et al. 2008; Forloni 2012). All of these aspects are components of building trust, which is critical to public engagement (Tannert et al. 2007; Berube et al. 2010).

Although avoiding hyperbole about hazards, risks, and benefits is important, it does not mean that in the face of uncertainty, immutable precautionary measures must be instituted. Rather, strict precautionary measures should be implemented when high levels of uncertainty about the potential health risk exist and then be modified as more scientific information becomes available.

If nanotechnology is to be fostered and its benefits realized, there is need to show that any health risks associated with exposure to nanomaterials can be minimized. A classic pitfall is the premise that putting one part of the population at risk can inversely provide a benefit to others (e.g., workers needlessly exposed to cotton dust in the production of cotton textile products) (American Textile Manufacturers Institute Inc. et al. versus Donovan, Secretary of Labor et al. 1981). This is contrary to the law and ethical presumptions in the United States and elsewhere. Maintaining the health and safety of the workforce while promoting development of nanotechnology can be in conflict and this must be guarded against, but since the two are linked, this linkage is promoted as an important criterion for responsible development. There cannot be responsible development of nanotechnology if workers are harmed.

The successful realization of the benefits of nanotechnology will be based, in part, on the public perception of risks and opinions of whether risk concerns are being addressed (Pidgeon et al. 2008). Although risk perception research focuses on “social risk phenomena” that are not covered in traditional risk assessment, such research needs to be an integral part of the effort in determining how the public reacts to workers’ risks and the efforts to control them (Harthorn 2006). It also should be noted that responsible development of nanotechnology involves many other factors, and ethical issues that are not derivative of worker risks (Alloff and Lin 2008).

Another aspect of responsible development of nanotechnology is the need to support research over the total life cycle of nanomaterials, so that occupational exposure to nanomaterials may not also lead to environmental exposures (Beaudrie et al. 2013). This life cycle focus may be an efficient use of resources and lead to a holistic assessment of the impact on people, organisms, and ecosystems (Karn and Bergeson 2009). Designing out the hazardous properties in nanomaterials may be a solution.

Responsible development of nanotechnology requires that society understand complex issues of hazard, exposure, dose, risk, and control as well as the potential for impact of nanotechnology on labor markets (Harthorn 2006; Pidgeon et al. 2008; Invernizzi 2011). This calls for achieving and maintaining a heightened level of scientific literacy and engagement (Bauer 2009). This will require continued and enhanced investment in education, training, and awareness across all ages and socioeconomic levels, including both workers and the general population, and it may be best to start with or include the K-12 population. The investment in education can also stimulate new generations of scientists to work on optimizing the benefits of nanotechnology.

Further, these efforts to protect workers and foster the benefits of nanotechnology must be based on a global vision, since the development, manufacture, and use of nanomaterials will be globally, albeit not evenly, distributed. Common global understanding of the elements of responsible development of nanotechnology is needed.

## Extent of compliance with precautionary guidance

In these opening decades of commercial nanotechnology, there are many examples showing that the principles and practices of responsible development have enjoyed broad support (Tomellini and Giordani 2008; NNI 2011; Forloni 2012; BIAC 2013). However, it is not clear to what extent precautionary guidance is being followed. This needs to be assessed on a national and global basis. Preliminary investigations have been a good start, but reflect small response rates and potential volunteer bias (ICON 2006; Engeman et al. 2012). More detailed and rigorous evaluations are required to minimize such bias. Plans are under way to develop such evaluations, but these efforts are expensive, and it will be difficult to identify and access employers and workplaces (Schulte and Iavicoli 2012; 78 Federal Register 2013). Business, government, labor, and other organizations must invest in developing and coordinating such evaluations. Assessing the extent to which there is compliance with precautionary guidance to protect workers involved with nanomaterials is a critical benchmark of responsible development of nanotechnology. Additionally, after such an evaluation is conducted, it will be important to identify hot spots, i.e., sectors, subsectors, and types of establishments or enterprises where compliance is less than appropriate and then institute remediation and strategic intervention (such as information campaigns).

## Conclusion

If the kinds of problems that have plagued previous emergent technologies are to be avoided, criteria are needed to define the responsible development of nanotechnology. The cornerstone of responsible development is the duty to protect workers, who are the first people exposed to the potential hazards of the technology. Protecting consumers and the environment are also important, but the foundation of responsible development begins with worker protection. However, these are not unrelated efforts. This article identifies five criterion actions that together can ensure responsible development of nanotechnology. All of these criteria are necessary components, and they need to be integrated with each other in practice, so that the knowledge gained through their implementation helps to advance the benefits of this technology. If these criteria are to be of value and applied, corporate and political support, globally, will be required. A lack of such support could pose a risk of harm to workers and could result in societal resistance to the development of nanotechnology.
